# Redox dysregulation as a link between childhood trauma and psychopathological and neurocognitive profile in patients with early psychosis

**DOI:** 10.1073/pnas.1812821115

**Published:** 2018-11-19

**Authors:** Luis Alameda, Margot Fournier, Ines Khadimallah, Alessandra Griffa, Martine Cleusix, Raoul Jenni, Carina Ferrari, Paul Klauser, Philipp S. Baumann, Michel Cuenod, Patric Hagmann, Philippe Conus, Kim Q. Do

**Affiliations:** ^a^Unit for Research in Schizophrenia, Center for Psychiatric Neuroscience, Department of Psychiatry, Lausanne University Hospital (CHUV), CH-1008 Prilly-Lausanne, Switzerland;; ^b^Service of General Psychiatry, Treatment and Early Intervention in Psychosis Program, Lausanne University Hospital (CHUV), CH-1008 Lausanne, Switzerland;; ^c^Signal Processing Laboratory, Ecole Polytechnique Fédérale de Lausanne, CH-1015 Lausanne, Switzerland;; ^d^Department of Radiology, Lausanne University Hospital (CHUV), CH-1011 Lausanne, Switzerland

**Keywords:** psychosis, early psychosis, childhood trauma, oxidative stress, psychopathology

## Abstract

Exposure to childhood trauma (CT) increases the risk for psychosis and affects the development of brain structures, possibly through oxidative stress. As oxidative stress is also linked to psychosis, it may interact with CT, leading to a more severe clinical phenotype. In 133 patients with early psychosis (EPP), we explored the relationships between CT and hippocampal, amygdala, and intracranial volume (ICV); blood antioxidant defenses [glutathione peroxidase (GPx) and thioredoxin/peroxiredoxin (Trx/Prx)]; psychopathological results; and neuropsychological results. Nonadjusted hippocampal volume correlated negatively with GPx activity in patients with CT, but not in patients without CT. In patients with CT with high GPx activity (high-GPx+CT), hippocampal volume was decreased compared with that in patients with low-GPx+CT and patients without CT, who had similar hippocampal volumes. Patients with high-GPx+CT had more severe positive and disorganized symptoms than other patients. Interestingly, Trx and oxidized Prx levels correlated negatively with GPx only in patients with low-GPx+CT. Moreover, patients with low-GPx+CT performed better than other patients on cognitive tasks. Discriminant analysis combining redox markers, hippocampal volume, clinical scores, and cognitive scores allowed for stratification of the patients into subgroups. In conclusion, traumatized EPP with high peripheral oxidation status (high-GPx activity) had smaller hippocampal volumes and more severe symptoms, while those with lower oxidation status (low-GPx activity) showed better cognition and regulation of GPx and Trx/Prx systems. These results suggest that maintained regulation of various antioxidant systems allowed for compensatory mechanisms preventing long-term neuroanatomical and clinical impacts. The redox marker profile may thus represent important biomarkers for defining treatment strategies in patients with psychosis.

## Significance

Early traumatic experiences interact with redox regulation and oxidative stress. Blood glutathione peroxidase (GPx) activity, involved in reducing peroxides, may reflect the oxidation status of the organism, thus allowing for the stratification of patients. Traumatized patients with psychosis who have a high blood oxidation status (high-GPx) have smaller hippocampal volumes (but not a smaller amygdala or intracranial volume), and this is associated with more severe clinical symptoms, while those with a lower oxidation status (low-GPx) showed better cognition and a correlated activation of the antioxidant thioredoxin/peroxiredoxin system. Thus, in patients with psychosis, traumatic experiences during childhood may interact with various redox systems, leading to long-term neuroanatomical and clinical defects. This redox profile may represent important biomarkers for patient stratification, defining treatment strategies at early stages of psychosis.

Exposure to childhood trauma (CT) in the form of child abuse and/or neglect is a major public health and social welfare problem worldwide, affecting 4–16% of children every year (1). CT increases vulnerability to a broad number of medical and major psychiatric conditions (2), including psychosis, where CT is now recognized as a major risk factor (3). Exposure to CT impacts the development of brain structures involved in psychosis, such as the hippocampus and amygdala (2, 4), and is associated with a poorer clinical profile in psychotic patients than in those without CT exposure (5). While some advances have been made in the understanding of the biological substrates underlying the link between CT and psychosis, the molecular mechanisms that mediate this diathesis remain unclear.

Genetic and developmental environment risks converging on oxidative stress as one central hub formed by neuroinflammation (6), NMDA receptor hypofunction (7), dopamine dysfunction, and redox dysregulation (8, 9) stand out as a potential pathophysiological mechanism of psychosis. This hub may play a mediating role in the link between the exposure to environmental insults and the later development of psychosis (6, 7, 10). In patients with schizophrenia, marks of oxidative stress and abnormal levels of antioxidant defenses were reported in peripheral samples as well as in patients’ brains (6–8, 11). In animal models, psychosocial insults at sensitive stages of brain development also lead to oxidative stress (6).

Redox processes form an adaptive system to respond to the environment that is required to maintain health in a changing environment (12). Antioxidant enzymes detoxify reactive oxygen species (ROS) into less reactive molecules, thus participating in redox homeostasis: Glutathione peroxidase (GPx) and peroxiredoxin (Prx) are two families of enzymes that catalyze the reduction of peroxides using the reducing power of glutathione (GSH) and thioredoxin (Trx), respectively. Oxidized GSH (GSSG) and oxidized Trx are, in turn, reduced by glutathione reductase (GR) and thioredoxin reductase (*SI Appendix*, Fig. S1). GSH, the most abundant endogenous nonprotein antioxidant, also scavenges ROS directly. GSH concentrations are decreased in blood (8, 13), cerebrospinal fluid (8, 14), and postmortem brain tissues (8, 15) of psychotic patients compared with those in healthy controls. In mice, reductions in GSH levels lead to neural connectivity impairments (16), and additional oxidative challenges during brain development impair neural synchronization (17), mimicking the deficits observed in schizophrenia. The Trx/Prx system is less well characterized than the GSH system in psychotic patients. A few studies reported an increase in plasma Trx levels in psychotic patients compared with those in healthy controls (18–20), which might be more pronounced in the acute phase of the illness (18, 20) and milder or absent in the chronic stage of the illness (18, 21), and one study reported an increase in brain Prx6 levels (22).

We previously reported that high blood GPx activity, a peripheral marker reflecting low brain GSH levels (23), is associated with reduced hippocampal volume in patients with early psychosis (EPP) (24), without taking into account a possible role of trauma exposure in such an association. Redox dysregulation, detected by high-GPx activity and reflecting high oxidation status, may underlie the decrease in hippocampal volume observed in traumatized patients (25).

The aim of the present study is to explore the relation between exposure to CT and the redox system in EPP. Toward this objective, we recruited EPP and collected brain imaging data (*n* = 64) as well as concomitant blood samples (*n* = 118) to quantify markers of GSH and Trx/Prx antioxidant systems.

## Results

### Demographics.

**Fig. 1.**
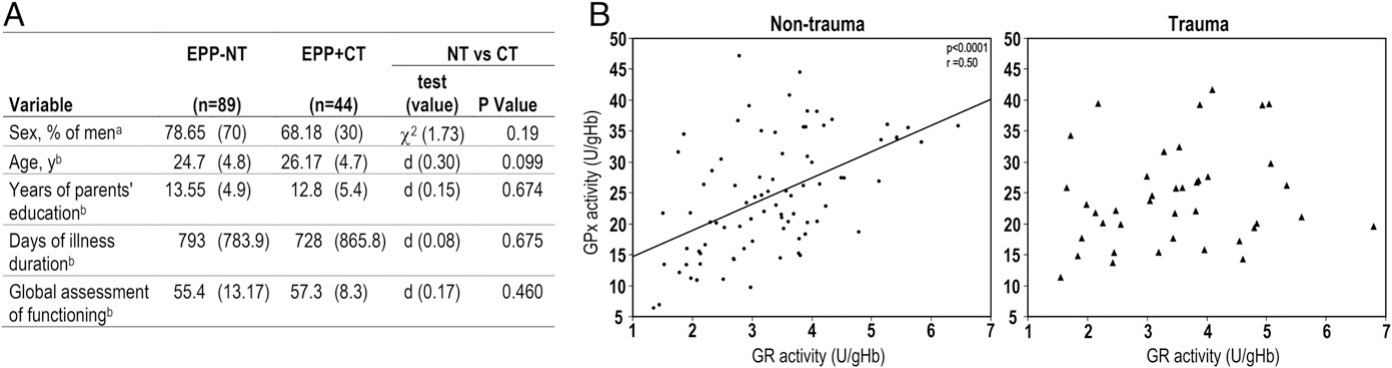
Alteration of redox homeostasis in blood of traumatized patients. (*A*) Demographic and clinical characteristics of EPP without trauma experience (EPP−NT) or with CT (EPP+CT). Data are presented as a percentage (*n*) (a) and as the mean (SD) (b). (*B*) Scatterplots illustrating the relation between GPx and GR activity [units per gram of Hb (U/gHb)] measured in hemolysates from EPP−NT (*Left*) and EPP+CT (*Right*). Pearson’s correlation coefficient indicated a positive correlation between GPx and GR activity in EPP−NT (*r* = 0.50, *P* < 0.0001). No correlation was detected in EPP+CT.

**Fig. 2.**
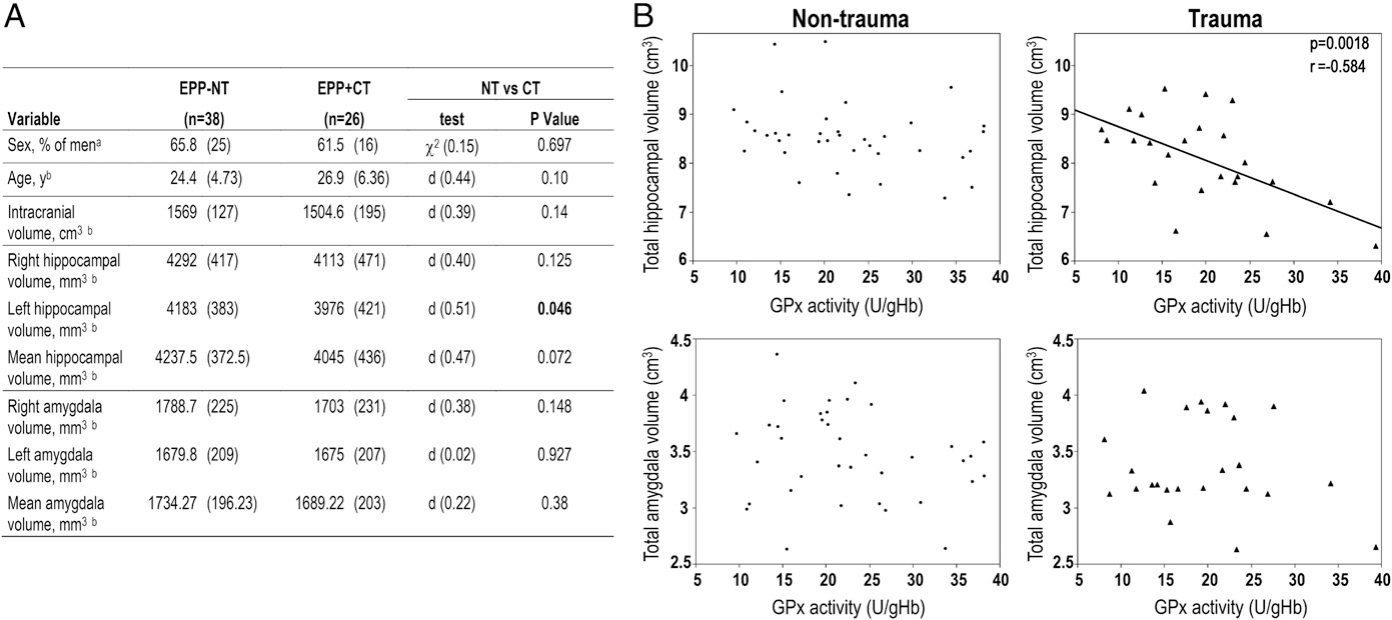
Smaller hippocampus is associated with a more oxidized status in blood of traumatized patients. (*A*) Demographic and anatomical characteristics of the subgroup of EPP with imaging scans. Data are presented as a percentage (*n*) (a) and as the mean (SD) (b). *P* < 0.05 is indicated in bold. (*B*) Scatterplots illustrating the relation between blood GPx activity [units per gram of Hb (U/gHb)] and total hippocampal volume (*Top*) or total amygdala volume (*Bottom*) in EPP−NT (*Left*) and EPP+CT (*Right*). Pearson’s correlation coefficient indicated a negative correlation between hippocampal volume and GPx activity in EPP+CT (*r* = 0.58, *P* = 0.0018). No correlation was detected in EPP–NT or with the amygdala.

Among 141 EPP recruited, eight were excluded for the following reasons: age at exposure to trauma was not available (*n* = 1), or first exposure to trauma occurred after psychosis onset (*n* = 3) or after the age of 16 y (*n* = 4). Analyses were carried out on the data of 133 patients (Fig. 1*A*), among whom 44 (33%) had a history of CT (EPP+CT). EPP+CT and EPP with no trauma exposure (EPP−NT) had similar age, sex, and socioeconomic status based on education of parents. The groups did not differ in terms of functioning, illness duration, or diagnostic breakdown (Fig. 1*A* and *SI Appendix*, Table S1). MRI scans were available for a subgroup of 64 patients: 38 EPP−NT and 26 EPP+CT matched for age and sex (Fig. 2*A* and *SI Appendix*, Table S2).

### GPx/GR Antioxidant System and Hippocampal Volume.

We tested whether EPP−NT and EPP+CT displayed a different redox status by assessing blood GPx and GR activities, two complementary enzymes of the GSH system (*SI Appendix*, Fig. S1). Mean activity of GPx and GR and mean concentrations of GSH were not different between the blood of EPP−NT and EPP+CT (*SI Appendix*, Table S1). In EPP−NT, we observed a positive correlation between GPx and GR activities (*r* = 0.50, *P* < 0.0001), suggesting a balanced oxidoreduction of the GSH system. This correlation was absent in traumatized patients, suggesting that trauma altered GSH homeostasis (Fig. 1*B*).

Compared with EPP−NT, EPP+CT had a smaller left hippocampal volume (*P* = 0.046) but a similar amygdala volume (*P* = 0.380; Fig. 2*A*).

To test whether redox dysregulation may explain this region-specific decrease in volume in EPP+CT, we assessed the correlation between GPx activity and hippocampal or amygdala volume in EPP−NT and EPP+CT (Fig. 2*B*). A negative correlation between blood GPx activity and hippocampal volume was observed in EPP+CT (*r* = −0.584, *P* = 0.0018) but not in EPP−NT. The same pattern was observed for the right and left hippocampus or when the ratio of GPx/GR, instead of GPx activity alone, was used (*SI Appendix*, Fig. S2). No correlation between blood GPx activity and amygdala volume was observed in EPP+CT or EPP−NT (Fig. 2*B*). Therefore, the association between higher oxidation status and smaller hippocampal volume in traumatized patients suggests that the interplay between CT and redox systems is region-specific.

As the association between trauma and smaller hippocampal volume was greater in patients with a high oxidative state (high-GPx activity), we stratified patients into the following four groups: (*i*) low-GPx EPP−NT, (*ii*) high-GPx EPP−NT, (*iii*) low-GPx EPP+CT, and (*iv*) high-GPx EPP+CT (*SI Appendix*, Table S2). The cutoff value between high- and low-GPx activity was chosen based on a recent add-on clinical trial with an antioxidant showing that GPx activity above 22.3 U/g of Hb predicted symptom improvement (26). High-GPx EPP+CT displayed significantly smaller hippocampal volumes than the three other groups (*P* = 0.020 and *P* = 0.035 for the comparisons of the right and left hippocampus, respectively, between high- and low-GPx EPP+CT; *SI Appendix*, Table S2). Interestingly, the mean volume of the hippocampus was similar between low-GPx EPP+CT and high- or low-GPx EPP−NT. We thus explored whether low-GPx EPP+CT benefited from compensatory mechanisms of other antioxidant systems, such as the Trx/Prx system (*SI Appendix*, Fig. S1).

### Stratification Based on Trauma and Antioxidant Profile.

#### Interaction between GPx/GR and Trx/Prx systems.

**Fig. 3.**
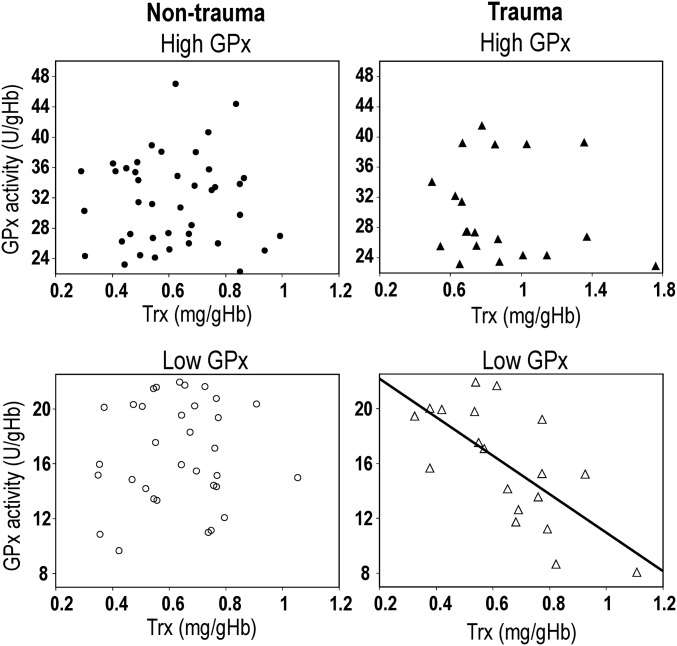
Compensatory regulation of the Trx/Prx system in trauma patients with low-GPx activity in blood. Scatterplots illustrate the relation between active Trx levels [milligrams per gram of Hb (mg/gHb)] and blood GPx activity [units per gram of Hb (U/gHb)] in EPP−NT (*Left*) and EPP+CT (*Right*) with high blood GPx activity (*Top*) and with low blood GPx activity (*Bottom*). Trx levels and GPx activities correlated negatively in low-GPx EPP+CT (*r* = −0.725, *P* = 0.0015). No correlations were detected in the other groups.

We investigated the relation between Trx levels and (*i*) GPx activity and (*ii*) oxidized Prx levels in the four groups of patients. A negative correlation between Trx levels and GPx activity was observed in low-GPx EPP+CT (*r* = −0.725, *P* = 0.0015) but not in the other groups (Fig. 3). Similarly, a negative correlation was also present between oxidized Prx levels and Trx levels only in low-GPx EPP+CT (*r* = −0.78, *P* = 0.0064; *SI Appendix*, Fig. S3).

Altogether, these results show that the profile of peripheral redox markers distinguished two groups of EPP+CT: (*i*) those with low-GPx activity, similar hippocampal volumes as EPP−NT, and compensatory regulation of the Trx/Prx system and (*ii*) those with high-GPx activity and smaller hippocampal volumes without compensation by the Trx/Prx system (*SI Appendix*, Fig. S3).

#### Psychopathological and neurocognitive profiles.

To challenge the validity of blood GPx activity as a criterion for patient grouping, we evaluated whether these biomarker-based patient subgroups presented different psychopathological and neurocognitive profiles.

**Fig. 4.**
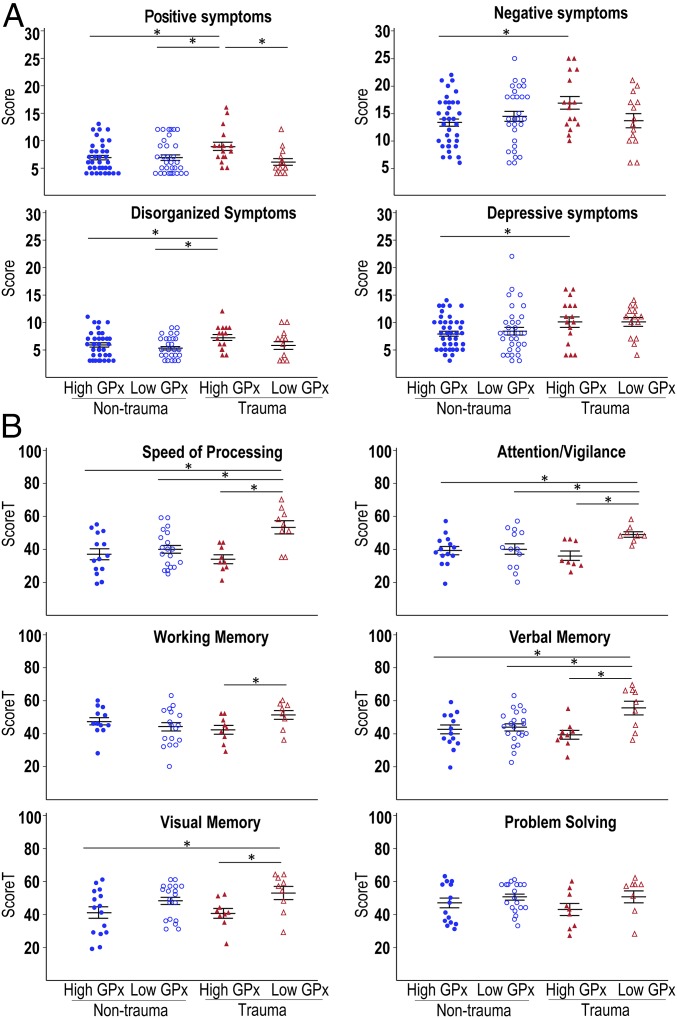
Psychopathological and neurocognitive profiles. (*A*) Symptoms were evaluated using the Positive and Negative Syndrome Scale in high- and low-GPx EPP−NT and high- and low-GPx EPP+CT. Dot plots illustrate individual scores, group mean, and SD for four subscales of the Wallwork et al. (51) five-factor model. (*B*) Neurocognition was assessed using the MATRICS Consensus Cognitive Battery. Dot plots illustrate individual standardized *t* scores, group mean, and SD for the six factors evaluated. *P < 0.05.

We compared the four groups of patients in terms of symptom severity and neurocognitive scores. Fig. 4*A* shows that high-GPx EPP+CT had higher levels of positive symptoms than the other three groups (*P* < 0.05) and higher levels of disorganized symptoms than EPP−NT (*P* < 0.05). High-GPx EPP+CT also showed higher levels of depressive and negative symptoms than high-GPx EPP−NT (*P* < 0.05). No differences were found between groups in the excited dimension.

For neurocognition, low-GPx EPP+CT performed significantly better than the other three groups in terms of speed of processing, attention/vigilance, and verbal memory (*P* < 0.05; Fig. 4*B*). They also had better scores in visual memory compared with high-GPx EPP−NT (*P* = 0.02) and high-GPx EPP+CT (*P* = 0.03) and better working memory compared with high-GPx EPP+CT (*P* = 0.046). No differences between groups were observed for problem solving.

#### Discriminant analyses.

Finally, we performed a classification analysis by linear discriminant analysis (LDA) to characterize and predict the group membership of each patient.

**Fig. 5.**
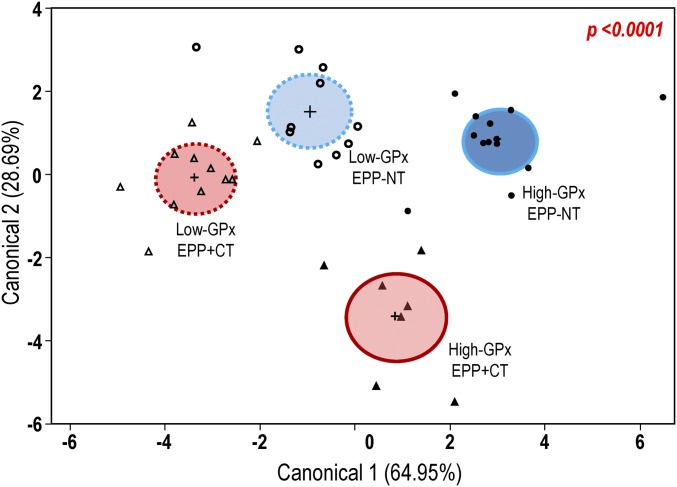
LDA was applied to the biochemical (GR activity, active Trx levels, and oxidized Prx levels), neuroanatomical (right and left hippocampal volume), and clinical (MATRICS Consensus Cognitive Battery *t* scores, positive symptoms, negative symptoms, disorganized symptoms, and depressive factors) data. The scatterplot diagram illustrates individual values of the first two canonical axes of the model, group means (+), and 95% confidence interval (ellipse). The canonical axes 1 and 2 of the model explained 64.95% and 28.69% of the variance, respectively. The four groups were significantly separated according to the four MANOVA tests: Wilks’ Lambda, Lawley’s trace, Roy’s largest root, and Pillai’s trace tests (*P* < 0.0001). EPP+CT, red; EPP−NT, blue; high-GPx, dark color; low-GPx, light color.

Variables included in the LDA model were redox markers (GR, Trx levels, and oxidized Prx), right and left hippocampal volume, and psychopathological and neurocognitive scores (*SI Appendix*, Table S3). The two first canonical axes explained 65% and 29% of variability. The scatterplot diagram illustrates that the four groups were significantly separated according to the four multivariate ANOVA (MANOVA) tests (*P* < 0.0001; Fig. 5).

In conclusion, LDA showed that the combination of redox markers and hippocampal volume efficiently distinguished two profiles of EPP+CT. The addition of clinical data further improved the classification, allowing for additional distinction between the profiles of the EPP−NT.

## Discussion

This study investigated the relation between exposure to CT and redox dysregulation in EPP, which are proposed to underlie schizophrenia pathophysiology. Our results showed that two distinct patient profiles can be defined on the basis of their redox status. On the one hand, EPP+CT with high-GPx activity, reflecting high oxidation status, displayed smaller hippocampal volumes and higher levels of positive and disorganized symptoms than EPP−NT. On the other hand, EPP+CT with low-GPx activity scored better than the other EPP on the speed of processing, verbal memory, and attention/vigilance neurocognitive tests. Considering that low-GPx EPP+CT showed a homeostatic regulation between the GPx/GR and Trx/Prx antioxidant systems, these results suggest that a preserved antioxidant balance contributes to the prevention of some of the neuroanatomical anomalies associated with trauma. A compensatory mechanism underlying the preserved regulation between the Trx/Prx and GPx/GR systems may occur in low-GPx EPP+CT, and therefore may prevent the accumulation of oxidation products, such as oxidized Prx. This compensatory mechanism was not present in high-GPx EPP+CT, and thus may underlie the hippocampal alterations observed in this group. Future studies may explore whether this mechanism also plays a role in nonpsychotic individuals exposed to trauma.

Some limitations should be considered. First, exposure to CT was determined retrospectively, which can be particularly problematic for patients suffering from psychosis due to recall bias (27). However, exposure to CT was assessed on the basis of information obtained from patients and their families in the context of a 3-y therapeutic relationship (28), which reduced the risk of recall bias that exists in other forms of self-report or cross-sectional research interviews. Moreover, trauma exposure and psychosis may share some risk factors. Second, the usual shortcomings linked to the relatively small sample size should also be considered. It precluded the stratification of patients according to other variables, such as age at trauma exposure or the repetition and length of exposure, which are known to modulate symptom severity (5), and may have had an impact on the neuroanatomical results. Third, the cutoff value for GPx activity, determined in a clinical trial involving a limited number of EPP (*n* = 61) (26), should be refined and must be tested in larger cohorts with prospective settings. Nevertheless, the discriminant analysis indicated that stratification based on GPx activity is a powerful approach to distinguish traumatized patients with different clinical and neuroanatomical profiles. Finally, we focused on the hippocampus as it is the most frequently studied stress-sensitive brain structure in the field of psychosis. The decrease in hippocampal volume observed in EPP+CT compared with EPP−NT was no more significant after correction by intracranial volume (ICV), suggesting that such a decrease in the hippocampus volume is related to an overall reduction of the ICV in traumatized patients (*SI Appendix*, Table S2), which, in turn, has been linked to cognitive deficits (29). However, we detected no effect on amygdala volume, suggesting that a specific interplay between trauma and redox occurs in the hippocampus. This could be explained by different maltreatment-sensitivity periods, which peak at the age of 3–5 y for the hippocampus and at the age 10–11 y for the amygdala (4). We previously showed in a large sample that EPP are more likely to be exposed to trauma in childhood than in adolescence (30). Nevertheless, our findings may also be applicable to other brain regions that have not yet been examined, and this possibility should be further explored.

CT may interact with the redox system through multiple mechanisms. Environmental stress triggers the secretion of stress-responsive hormones, including glucocorticoids (31), through hypothalamic–pituitary–adrenal (HPA) axis activation. Feedback loops then inhibit the system, favoring a return to homeostatic levels. However, this regulation is impaired if stress exposure persists for months, as shown by human and primate studies reporting alterations of HPA axis activation in children exposed to severe life stress (31), and glucocorticoids induced by acute stress may actually prime neuroinflammation to subsequent challenges (32, 33). A meta-analysis showed that CT is associated with a proinflammatory state in adulthood (34). Severe life stress is also associated with brain oxidative stress (35). Interestingly, Trx interacts directly with the DNA-binding domain of the glucocorticoid receptor under oxidative conditions (36). In parallel, epigenetic changes may mediate the long-lasting effect of CT and the interplay between early adversity and the development of mental diseases (37). In the mouse, glucocorticoid treatment induced epigenetic modification of similar pathways in the blood and in the brain (38). This study thus supports the use of whole blood to identify glucocorticoid-induced brain changes. Conversion to psychosis is associated with epigenetic changes in redox genes (9); therefore, it would be interesting to examine at a genome-wide level whether the abnormalities that we observe at a clinical, biological, and neuroanatomical levels in traumatized EPP are related to changes in the methylation status of genes implicated in the redox system.

Our data indicate that EPP who were exposed to trauma can be split into two subgroups: one with a severe clinical phenotype and oxidized state, reflected by high blood GPx activity, and one with better functioning, characterized by a homeostatic regulation involving the Trx/Prx antioxidant system. Further investigations on mechanisms underlying the regulation between the GPx/GR and Trx/Prx systems is warranted. In this regard, it is interesting to note that Trx prevents the inhibition of the glucocorticoid receptor by oxidative stress and preserves the expression of genes induced by the glucocorticoid receptor, a mechanism thought to prevent the overshoot of inflammation (36, 39). This coordination of the HPA axis-mediated stress response and cellular redox system may not be preserved in patients with impaired Trx regulation, leading to more sustained activation of inflammation.

The interplay between inflammation and oxidative stress is well established, and their reciprocal activation can lead to feed-forward deleterious processes underlying brain alterations (40). Fast-spiking parvalbumin interneurons are particularly vulnerable to redox imbalance/oxidative stress due to their high frequency of discharge, which implies enhanced oxidative metabolism activity (6, 41). Impairments of these interneurons, present in both patients with schizophrenia and models, play a critical role in neural synchronization and cognitive deficits. Genetic and environmental risks appear to converge on oxidative stress-induced parvalbumin interneuron impairments (6). Therefore, trauma experience, through overactivation of the HPA axis, may contribute to the exacerbation of the vicious cycle between oxidative stress and neuroinflammation. In patients with impaired redox regulation who are exposed to trauma, these mechanisms may act synergistically to impair parvalbumin interneurons and lead to a severe phenotype.

Clinically, no consensus on the treatment of the sequelae of trauma in psychotic patients has been reached. There is a need to identify markers that may help clinicians to select candidates with greater potential for improvement under specific therapeutic interventions. We show that EPP+CT with low-GPx activity perform better than the other groups in three neurocognitive tasks, with scores that are within the normal range or above. These findings may have some clinical implications. The preserved cognitive functions in this group make them good candidates for trauma-focused interventions (42). In contrast, traumatized patients with high-GPx levels, who present cognitive functions in the lower range of the mean, might be good candidates for cognitive remediation therapy (CRT). Additionally, CRT has been found to be more efficient in combination with other approaches (43). Therefore, supplementing CRT with antioxidant compounds in EPP with a disrupted redox homeostasis may help to improve their cognition (26), and subsequently enhance their functional level.

Previous studies have already reported that EPP exposed to trauma suffer from higher levels of positive symptoms than nonexposed patients (5, 44). Our data show that this is true mostly for patients displaying redox dysregulation. This highlights that the GPx/GR redox system, together with trauma exposure status, is a marker for a poorer psychopathological profile in EPP. Outcome prediction with biomarkers has been the challenge of research in psychiatry in the past 20 y. Our study brings an important contribution to the field by highlighting a stratification of patients with different psychopathological and neurocognitive profiles based on a combination of demographic (trauma exposure status) and biological peripheral markers (GPx/GR and Trx/Prx systems) at the beginning of their treatment. This biomarker-based classification is a promising approach to refine specific treatments strategies in the early stage of psychosis.

## Materials and Methods

### Participants.

Patients were recruited from the Treatment and Early Intervention in Psychosis Program (TIPP-Lausanne), which offers 3 y of treatment to patients aged 18–35 y (45). Inclusion criteria were (*i*) meeting the psychosis threshold as defined by the Comprehensive Assessment of At-Risk Mental States (46), (*ii*) no antipsychotic medication for >6 mo, (*iii*) no psychosis related to intoxication or organic brain disease, and (*iv*) intelligence quotient ≥70. The diagnosis and the date of the psychosis threshold were determined following expert consensus between a senior psychiatrist and a senior psychologist who reviewed the entire files of patients and based on the *Diagnostic and Statistical Manual of Mental Disorders, Fourth Edition* (47). Duration of illness was defined as the time that elapsed between reaching the psychosis threshold for the first time and the time of assessment. All subjects who participated in this study provided informed written consent in accordance with our institutional guidelines (study and consent protocols were approved by the Ethical Committee of Lausanne University). Blood sampling, clinical assessment, and MRI imaging (when applicable) were concomitant.

### MRI Acquisition and Analysis.

MRI sessions were performed on a 3-T scanner (Siemens Medical Solutions) equipped with a 32-channel head coil. The hippocampal volumes and ICV were automatically segmented using FreeSurfer software, version 5.0.0 (48). Hippocampal volumes and ICV were analyzed independently, without normalization. FreeSurfer has demonstrated high reproducibility and consistency in hippocampus segmentation compared with manual tracing (48) (more details are provided in *SI Appendix*).

### Blood GPx Activity and Oxidized Prx and Trx Levels.

GPx and GR enzymatic activity in hemolysates was determined as previously described and expressed in enzymatic units per gram of hemoglobin (23). Prx and oxidized Prx levels in hemolysates were quantified by Western blot using the following primary antibodies: anti-2Cys Prx mouse (1:1,000 dilution, ab16765; Abcam) and anti-SO3-Prx (oxidized Prx) rabbit (1:2,000 dilution, ab16830; Abcam). Trx levels were assessed in hemolysates by end-point measurement of Trx reducing activity, using insulin as a substrate, with an adapted version of Arnér and Holmgren’s (49) protocol (details are provided in *SI Appendix*).

### Psychopathological and Neurocognitive Measures.

Psychopathological and neurocognitive measures were assessed by trained psychologists in face-to-face interviews. The level of symptoms was evaluated with the 30 items of the Positive and Negative Syndrome Scale (50). We used the Wallwork et al. (51) five-factor model of psychosis to categorize the positive, negative, disorganized/concrete, excited, and depressed dimensions. Neuropsychological assessments were administered with the MATRICS Consensus Cognitive Battery (52), excluding the Mayer–Salovey–Caruso Emotional Intelligence Test because the French translation had not been validated at the time of the study. Thus, nine of 10 subtests were given, comprising six factors: processing speed, sustained attention, working memory, verbal learning, visual learning, and problem solving. Scores were adjusted for age and sex.

### Assessment of History of Past Trauma.

Patients were considered traumatized if they were exposed to at least one experience of abuse (physical, sexual, or emotional) or neglect (physical or emotional), as these experiences have been shown to increase the risk for psychosis (3) and impact the psychopathological profile of EPP (3, 5). Patients were excluded if they were exposed to trauma after the age of 16 y or were in the prodromal phase of the disease when trauma occurred (also *SI Appendix*).

### Statistical Analysis.

Statistical analyses were performed using JMP software (JMP IN, version 12.1; SAS). All used variables passed the Shapiro–Wilk test for normal distribution. Using least squares analyses, we found no interaction between the variables of interest and age or sex, which were therefore not included in our models. For correlation analysis between variables of interest, we used the Pearson coefficient. For group comparisons, we performed one-way ANOVA, followed by the Student’s *t* test. We used LDA as a multivariate analysis to integrate the different variables into one model. Differences between groups were tested using MANOVA tests (Wilks’ Lambda, Lawley’s trace, Roy’s largest root, and Pillai’s trace tests; details on LDA are provided in *SI Appendix*). For all tests, we used 0.05 as the significance threshold for the *P* value.

## Supplementary Material

Supplementary File
