# Supplementary material for: Redox dysregulation as a link between childhood trauma and psychopathological and neurocognitive profile in patients with early psychosis
Source: Proc Natl Acad Sci U S A. 2018 Nov 19;115(49):12495–500. doi: 10.1073/pnas.1812821115 (PMC6298080; doi:10.1073/pnas.1812821115)
Supplement: Supplementary File [file pnas.1812821115.sapp.pdf]

SUPPORTING INFORMATION

Supporting Methods

Blood GPx activity and oxidized Prx and Trx levels

Morning blood was collected after fasting as of the previous midnight in EDTA tubes. Whole blood was immediately frozen at -80° to subsequently assess GSH as previously described. In parallel, blood was immediately centrifuged at 3,000 g for 5 min at 4°C. The pellet, corresponding to blood cells, was washed 2 times with 0.9% NaCl and frozen at -80°C. For analyses, hemolysates were prepared by dilution of blood cells at 1:10 in water.

To quantify Prx and oxidized Prx levels, hemolysates (10 µg of hemoglobin) were loaded on 12.5% acrylamide gels for SDS-PAGE and transferred to nitrocellulose membranes. Membranes were blocked in Tris Buffer Saline with 2.5% non-fat dried milk and hybridized overnight at 4° with a primary antibody diluted in blocking buffer with 0.1% Tween (anti-2Cys Prx mouse, dilution 1:1000 Abcam ab16765; anti-SO3-Prx (oxidized Prx) rabbit, dilution 1:2000 Abcam ab16830). Immunoblots were revealed using appropriate secondary antibodies coupled to infrared fluorescent dyes (anti-mouse IgG-800CW, Biosciences, 926-32210; anti-rabbit IgG-680RD, Biosciences, 926-68071). Images were acquired and quantified using the Odyssey imaging system (Li-cor Bioscience).

Trx levels were assessed by end-point measurement of Trx reducing activity, using insulin as the substrate, with an adapted version of Arnér & Holmgren's protocol(66). Hemolysates were pre-incubated with 100 mM DTT for 15 min at 37° to activate Trx (dilution 2.8). In wells of 96-well plates, this reaction was mixed either with the complete working solution (85 mM HEPES, 0.3 mM insulin, 660 µM NADPH, 3 mM EDTA, 50 nM TrxR) to assess total reducing activity or with the incomplete working solution (85 mM HEPES, 0.3 mM insulin, 660 µM NADPH, 3 mM EDTA) to assess the non-Trx-specific reducing activity. Reactions were incubated at 37° for 1 h and

www.pnas.org/cgi/doi/10.1073/pnas.1812821115

stopped by the addition of 10% DTNB with 5.4 M guanidine. Absorbance was read at 412 nm (Tecan). The mean of six replicates was calculated, the non-specific activity was subtracted from the total reducing activity, and the quantity of Trx in test samples was calculated based on a standard curve of recombinant Trx; Trx levels in test samples were normalized to hemoglobin content.

Assessment of history of past trauma

Clinicians were trained to conduct an extensive assessment of patients, including an evaluation of exposure to traumatic life events. Information related to exposure to traumatic experiences was collected by case managers (each patient was assessed by the same case manager during the three years of follow-up), who must fill in a table where exposure to different life events is recorded, including experiences of abuse (sexual, physical and emotional) and neglect (physical and emotional). This investigation was not based on a single interview, questionnaire, or self-report, but information was obtained within the framework of a trusting relationship that developed progressively during the treatment period. Case managers met with patients frequently over the treatment period, where extensive knowledge of patients' history was gathered. If patients agreed and if it was pertinent, information was also collected from family. In a case of inconsistency between information obtained from the family and the patient's information or in a case of doubt about the exposure or about the age of exposure and without any other source of verification (e.g., register from police or records from youth protection services), this patient was not included in the study. Exposure to traumatic life events was recorded as follows: i) Type of traumatic life event, rated as present or absent (sexual abuse, physical abuse, emotional and physical neglect, emotional abuse, among others); ii) time of first occurrence in relation

to psychosis stage (during the premorbid phase, during the prodromal phase, after psychosis onset); iii) age at the time of first exposure; iv) single or repeated exposure to each trauma.

Physical abuse refers to physical attack or assault or being repeatedly beaten by parents, relatives, or caregivers. Emotional abuse was defined as verbal assaults on a child's sense of worth or well-being or any humiliating or demeaning behavior directed toward a child by an adult or older person. Physical neglect was defined as the failure of caretakers to provide for a child's basic physical needs, including food, shelter, clothing, safety, and health care. Emotional neglect was defined as the failure of caretakers to meet children's basic emotional and psychological needs, including love, belonging, nurturance, and support.

MRI acquisition and analysis

The scanner was upgraded during the course of the study; so, 48% (n=31) of the scans were conducted on the Magnetom TrioTim, and 52% (n=33) on the Prisma system. The same acquisition protocol was used for both TrioTim and Prisma imaging, ensuring consistency of volumetric measures (1, 2) (Supporting Figure 4). Moreover, the results from analyses repeated on data from the TrioTim system were consistent with the results from analyses on the whole dataset (Supporting Figure 4B). Each scanning session included a magnetization-prepared rapid acquisition gradient echo (MPRAGE) T1-weighted sequence with 1-mm in-plane resolution and 1.2-mm slice thickness, covering 160x240×256 voxels. The repetition (TR), echo (TE) and inversion (TI) times were, respectively, 2300, 2.98 and 900 ms; the flip angle was 9 degrees. All images were visually inspected for artifacts or structural

abnormalities and were segmented using the FreeSurfer software (version 5.0.0. https://surfer.nmr.mgh.harvard.edu/)(3).

Statistical analysis

The LDA procedure selects factors that best predict group membership by reducing the variance within each group and maximizing the variance between groups(4). LDA provides a linear combination of the variables and finds the axes that maximize the distances between groups. Loadings of the canonical variables are presented in Supporting Table 3.The canonical plot illustrates the 2 canonical axes (CA1, CA2) that provided the best group discrimination. Differences between groups were tested using Multivariate Analysis of Variance tests, which tests the null hypothesis of equal mean vectors (i.e., of group overlap).

Supporting Tables, Figures, and Legends

Variable	EPP all (n=133)	EPP-NT (n=89)	EPP+CT (n=44)	NT vs CT test (value)	NT vs CT P Value
Sex, % of men[a]	75.18 (100)	78.65 (70)	68.18 (30)	χ^2 (1.73)	0.19
Age, y[b]	25.43 (4.75)	24.7 (4.8)	26.17 (4.7)	d (0.30)	0.099
Years of parents' education[b]	13.17 (5.15)	13.55 (4.9)	12.8 (5.4)	d (0.15)	0.674
Days of illness duration[b]	760.5 (824.8)	793 (783.9)	728 (865.8)	d (0.08)	0.675
Global assessment of functioning[b]	56.35 (10.73)	55.4 (13.17)	57.3 (8.3)	d (0.17)	0.460
Diagnoses				χ^2 (4.78)	0.442
Schizophrenia	60.1 (80)	65.2 (58)	50.0 (22)		
Schizophreniform/BPE	13.5 (18)	13.5 (12)	13.7 (6)		
Schizoaffective disorder	11.3 (15)	9.0 (8)	15.9 (7)		
Major depression	3.8 (5)	2.2 (2)	6.8 (3)		
Bipolar disorder	4.5 (6)	3.4 (3)	6.8 (3)		
Others	6.8 (9)	6.7 (6)	6.8 (3)		
GPx activity, U/gHb[b]	24.76 (9.32)	24.70 (9.11)	24.83 (9.54)	d (0.01)	0.942
GR activity, U/gHb[b]	3.39 (1.31)	3.43 (1.20)	3.36 (1.42)	d (0.05)	0.788
Blood GSH, umol/mL[b]	0.81 (0.24)	0.80 (0.24)	0.82 (0.25)	d (0.08)	0.787

Supporting Table 1

Demographic, clinical, and biochemical characteristics of early psychosis patients (EPP) without trauma experience (EPP-NT) or with childhood trauma (EPP+CT). [a]: data are presented as percentage (n); [b]: data are presented as mean (standard deviation). BPE: brief psychotic episode.

Variable	EPP-NT		EPP+CT		EPP+CT, high GPx vs EPP+CT, low GPx	
	high GPx (n=18)	low GPx (n=20)	high GPx (n=8)	low GPx (n=18)	test	P Value
Right hippocampal volume, mm^3	4158 (352)	4463 (370)	3804 (479)	4251 (408)	d (1.00)	**0.020**
Left hippocampal volume, mm^3	4090 (344)	4267 (405)	3727 (447)	4087 (370)	d (0.78)	**0.035**
Intracranial Volume, cm^3	1508 (110)	1624 (117)	1422 (165)	1552 (155)	d (0.81)	**0.066**
GPx activity	29.7 (5.9)	16.46 (3.9)	27.8 (5.9)	15.84 (4.2)	d (2.33)	**<0.0001**
Trx levels	0.61 (0.17)	0.64 (0.15)	1.02 (0.43)	0.63 (0.21)	d (1.15)	0.0002
oxidized Prx levels	0.019 (0.007)	0.014 (0.008)	0.015 (0.007)	0.024 (0.016)	d (0.72)	0.052

Supporting Table 2

Neuroanatomical characteristics and blood redox markers in the subgroup of EPP patients with imaging scans. Data are presented as mean (SD); P-Values < 0.05 are indicated in bold.

	GR (U/gHb)	Trx levels (ng/ug Hb)	left hipp. volume (mm3)	right hipp. volume (mm3)	Processing speed (Tscore)	Sustained attention (Tscore)	Working memory (Tscore)	Verbal learning (Tscore)	Visual learning (Tscore)	Problem solving (Tscore)	PANSS positive factor	PANSS negative factor	PANSS disorg. factor	PANSS excited factor	PANSS depresse d factor
**Total Canonical Structure**															
**Canon 1**	-0.0648	-0.0069	-0.0647	-0.1982	-0.2235	-0.1310	-0.0446	-0.1429	-0.4174	-0.2128	0.1740	-0.1936	0.0846	-0.0732	-0.1544
**Canon 2**	0.1397	-0.6044	0.5862	0.5477	0.1753	0.2433	0.1103	0.0615	0.1410	0.2121	0.1520	0.0906	-0.1437	-0.0756	-0.2954
**Canon 3**	0.4628	-0.3378	0.1010	-0.0865	0.0183	-0.3151	0.2973	-0.0060	0.0318	-0.1298	-0.2082	-0.3040	-0.0275	0.3581	0.0245
**Between Canonical Structure**															
**Canon 1**	-0.1868	-0.0114	-0.1170	-0.3608	-0.8073	-0.3873	-0.1930	-0.9283	-0.9529	-0.7016	0.6560	-0.6546	0.5311	-0.2770	-0.4895
**Canon 2**	0.3740	-0.9252	0.9853	0.9265	0.5885	0.6684	0.4429	0.3710	0.2992	0.6500	0.5325	0.2846	-0.8391	-0.2660	-0.8704
**Canon 3**	0.9084	-0.3792	0.1244	-0.1073	0.0449	-0.6350	0.8756	-0.0266	0.0495	-0.2918	-0.5349	-0.7004	-0.1176	0.9233	0.0529
**Pooled Within Canonical Structure**															
**Canon 1**	-0.0242	-0.0030	-0.0267	-0.0816	-0.0818	-0.0488	-0.0162	-0.0510	-0.1617	-0.0784	0.0635	-0.0712	0.0302	-0.0267	-0.0571
**Canon 2**	0.0730	-0.3628	0.3384	0.3154	0.0897	0.1267	0.0558	0.0307	0.0764	0.1093	0.0775	0.0466	-0.0718	-0.0386	-0.1527
**Canon 3**	0.3770	-0.3161	0.0909	-0.0777	0.0146	-0.2559	0.2346	-0.0047	0.0268	-0.1043	-0.1656	-0.2437	-0.0214	0.2847	0.0198
**Scoring Coefficients**															
**Canon 1**	-0.6190	0.0611	0.0037	-0.0035	-0.0244	0.0553	-0.0210	0.0328	-0.0284	-0.0624	0.2343	-0.0734	-0.0543	0.0007	0.0281
**Canon 2**	-0.7198	-3.8058	0.0014	0.0042	0.0095	0.1122	-0.1009	-0.0160	0.0092	-0.0349	-0.0652	0.0734	-0.1409	0.1573	0.0552
**Canon 3**	0.5789	-0.0858	0.0009	-0.0014	0.0020	-0.0980	0.1258	-0.0032	-0.0408	-0.0027	-0.0599	-0.0126	0.0308	0.1293	-0.1345
**Standardized Scoring Coefficients**															
**Canon 1**	-0.9377	0.0152	1.1818	-1.2523	-0.3150	0.6376	-0.2125	0.3923	-0.2910	-0.6867	0.7782	-0.4340	-0.1304	0.0018	0.1024
**Canon 2**	-1.0905	-0.9467	0.4306	1.5083	0.1228	1.2939	-1.0183	-0.1908	0.0947	-0.3837	-0.2165	0.4344	-0.3381	0.4054	0.2012
**Canon 3**	0.8770	-0.0214	0.2702	-0.4991	0.0256	-1.1305	1.2696	-0.0381	-0.4179	-0.0295	-0.1990	-0.0747	0.0739	0.3332	-0.4901

Supporting Table 3

Canonical details for linear discriminant analysis used in Figure 5

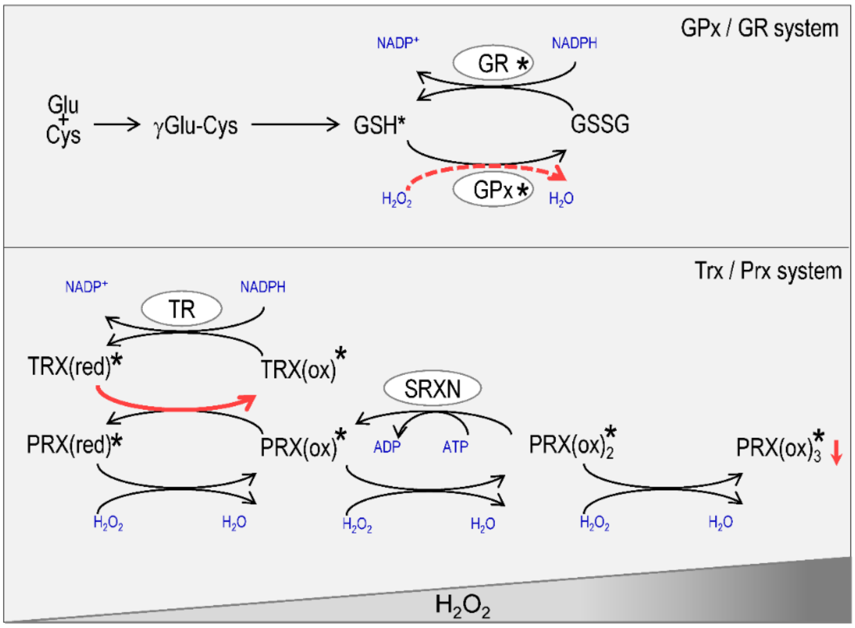

Supporting Figure 1

GPx/GR and Trx/Prx antioxidant systems. Illustration of the key players in the GPx/GR antioxidant system (upper part) and Trx/Prx antioxidant system (lower part). *: species or activities quantified in the study. In the text, Prx refers to all Prx forms described here (red, ox, ox_2, ox_3); oxidized Prx refers to $PRX(ox)_3$. Red arrows illustrate that in Low-GPx EPP+CT, lower GPx activity was associated with higher Trx activity and less accumulation of irreversibly oxidized $Prx(ox)_3$. In High-GPx EPP+CT, these associations were not observed. These patients with high GPx activity had a smaller hippocampal volume and more severe symptoms than those with low GPx activity.

GPx: glutathione peroxidase; GR: glutathione reductase; SRXN: sulfiredoxin; TR: thioredoxin reductase; TRX: thioredoxin; PRX: peroxiredoxin; (red): reduced form; (ox), $(ox)_2$, $(ox)_3$: oxidized forms i.e., sulfenic, sulfinic, and sulfonic, respectively. Note that PRX(ox)3 is irreversibly oxidized. Blue: cofactors. Names of the enzymes are circled.

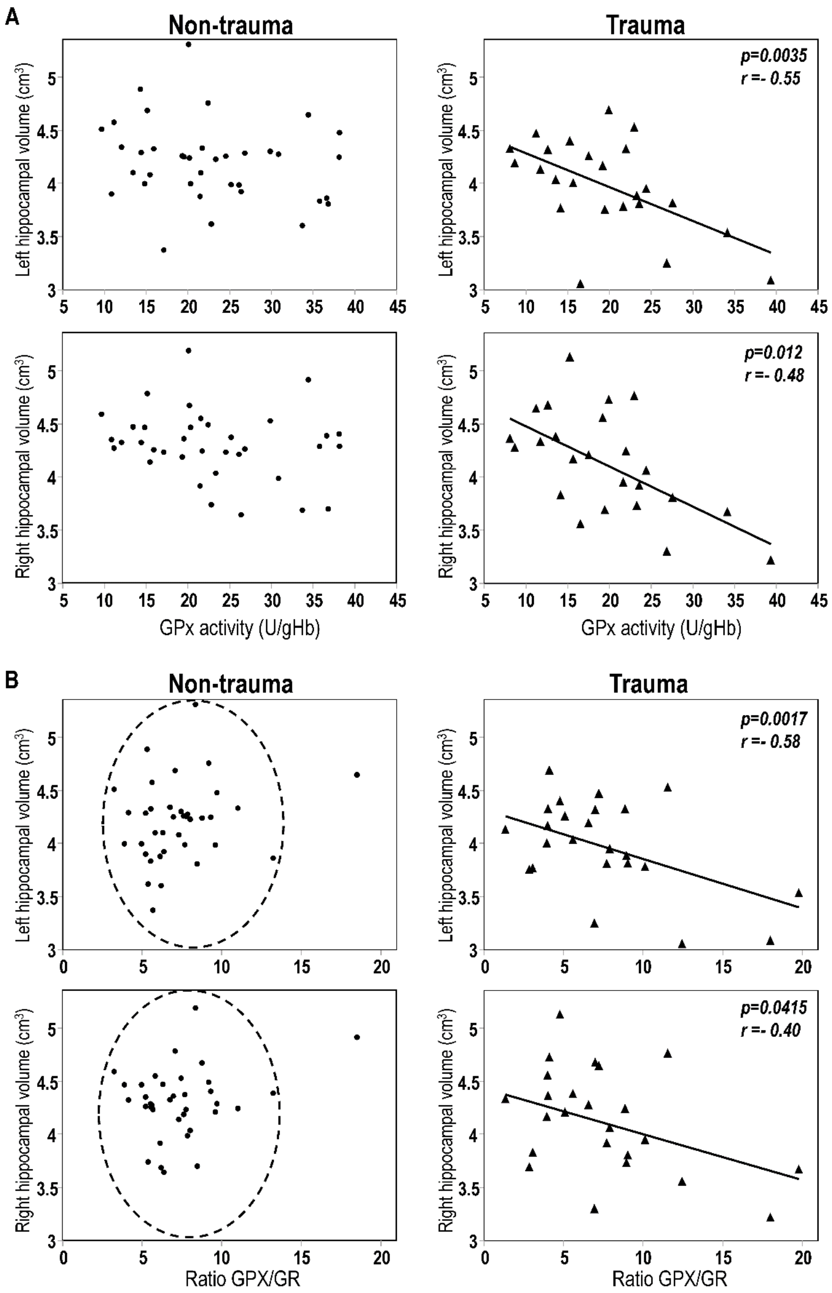

Supporting Figure 2

Smaller hippocampus is associated with a more oxidized status in blood. A) Scatterplots illustrating the relation between blood GPx activity (U/gHb) and left (upper row) or right (lower row) hippocampal volume. Pearson's correlation coefficients indicated a negative correlation between GPx activity and hippocampal volume in EPP+CT (left: r=-0.55; p=0.0035; right: r=-0.48; p=0.012). No correlation was detected in EPP-NT. **B)** Scatterplots illustrating the relation between the ratio of

GPx/GR activities and left (upper row) or right (lower row) hippocampal volume. Pearson's correlation coefficient indicated a negative correlation between the GPx/GR ratio and hippocampal volume (left: r=-0.58; p=0.0017; right: r=-0.40; p=0.041). No correlation was detected in EPP-NT.

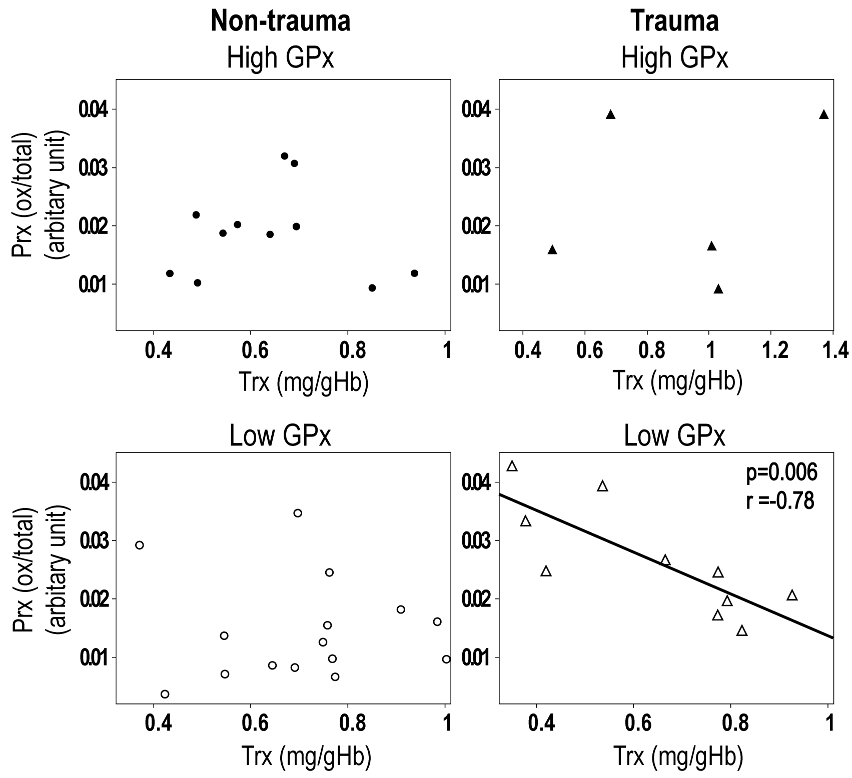

Supporting Figure 3.

Compensatory regulation of the Trx/Prx system in trauma patients with low GPx activity in blood. Scatterplots illustrating the relation between active Trx levels (mg/gHb) and the proportion of oxidized Prx over total Prx (Prx(ox/total)). Trx levels and the proportion of oxidized Prx correlated negatively in Low-GPx EPP+CT (r=-0.78p=0.006). No correlations were detected in the other groups.

A

Total hippocampal volume (mm³)

	Number	Mean	Std Dev
Prisma EPP-NT	19	8750.62	646.840
Prisma EPP+CT	14	8229.27	856.350
Trio EPP-NT	19	8179.68	768.178
Trio EPP+CT	12	7750.75	781.053

Total Amygdala volume (mm³)

	Number	Mean	Std Dev
Prisma EPP-NT	19	3398.13	420.803
Prisma EPP+CT	14	3336.97	373.580
Trio EPP-NT	19	3471.00	426.734
Trio EPP+CT	12	3292.50	385.723

B

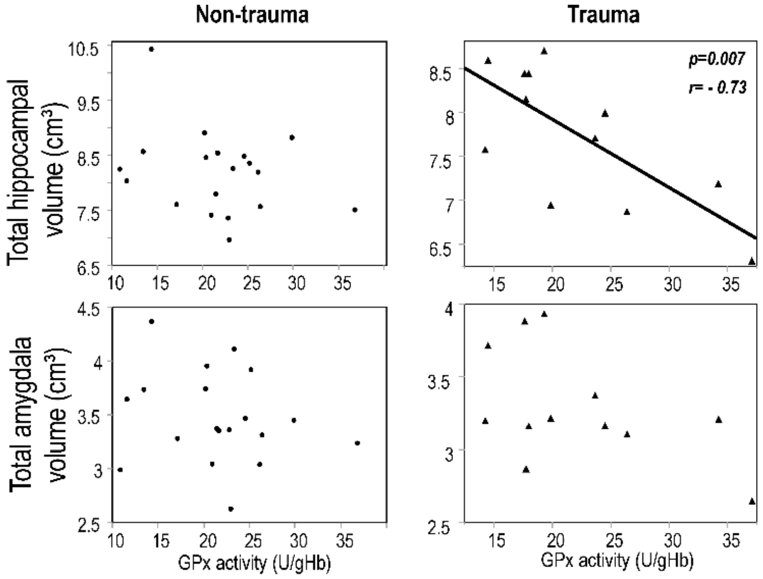

Supporting Figure 4

Comparison of 3-Tesla MRI scanners. A) Hippocampal and amygdala volumes measured by Prism or TrioTim systems; number indicates the n of patients scanned in each corresponding condition. **B)** Scatterplots illustrating the relation between GPx activity and hippocampal (upper row) or amygdala (lower row) volumes measured with the TrioTim scanner only. Pearson's correlation coefficient indicated a negative correlation between GPx activity and hippocampal volume in EPP+CT (r=-0.73; p=0.007). No correlation was detected for amygdala volume or in EPP-NT.

Supporting References

1. Mair R & McMains S (2017) A within-subject comparison of anatomical and diffusion scans from Siemens TimTrio and Prisma scanners *International Symposium on Magnetic Resonance in Medicine (ISMRM) 2017* #3923.
2. Cannon TD*, et al.* (2014) Reliability of neuroanatomical measurements in a multisite longitudinal study of youth at risk for psychosis. *Human Brain Mapping* 35(5):2424-2434.
3. Fischl B*, et al.* (2002) Whole brain segmentation: automated labeling of neuroanatomical structures in the human brain. *Neuron* 33(3):341-355.
4. Stewart S, Ivy MA, & Anslyn EV (2014) The use of principal component analysis and discriminant analysis in differential sensing routines. *Chemical Society Reviews* 43(1):70-84.
